# Insights into cognitive decline in spinocerebellar Ataxia type 2: a P300 event-related brain potential study

**DOI:** 10.1186/s40673-019-0097-2

**Published:** 2019-03-04

**Authors:** Roberto Rodríguez-Labrada, Luis Velázquez-Pérez, Ricardo Ortega-Sánchez, Arnoy Peña-Acosta, Yaimeé Vázquez-Mojena, Nalia Canales-Ochoa, Jacqueline Medrano-Montero, Reidenis Torres-Vega, Yanetza González-Zaldivar

**Affiliations:** 1 Centre for the Research and Rehabilitation of Hereditary Ataxias, Libertad Street # 26, 80100 Holguín, Cuba; 2 Faculty of Sport and Physical Culture, University of Holguín, Holguín, Cuba; 3 Cuban Academy of Science, street no. 460, Habana Vieja, La Habana, Cuba; 4 Medical University of Holguín, Lenin Avenue 1, Holguín, Cuba

**Keywords:** Spinocerebellar ataxia type 2, P300, Event–related evoked potentials, Biomarkers, Cognitive decline

## Abstract

**Background:**

Cognitive decline is a common non-motor feature characterizing Spinocerebellar Ataxia type 2 (SCA2) during the prodromal stage, nevertheless a reduced number of surrogate biomarkers of these alterations have been described.

**Objective:**

To provide insights into cognitive dysfunction in SCA2 patients using P300 event-related potentials (ERP) and to evaluate these measures as biomarkers of the disease.

**Methods:**

A cross-sectional study was performed with 30 SCA2 patients, 20 preclinical carriers and 33 healthy controls, who underwent visual, auditory P300 ERPs, and neurological examinations and ataxia scoring.

**Results:**

SCA2 patients showed significant increase in P300 latencies and decrease of P300 amplitudes for visual and auditory stimuli, whereas preclinical carriers exhibit a less severe, but significant prolongation of P300 latencies. Multiple regression analyses disclosed a significant effect of SARA score on visual P300 abnormalities in patients as well as of the time to ataxia onset on visual P300 latencies in preclinical carriers.

**Conclusions:**

This paper demonstrated the role of P300 ERP for the study of attentional, discriminative and working memory abnormalities in SCA2 patients and for the search of surrogate biomarkers from prodromal to the symptomatic stages. Moreover, our findings provide psychophysiological evidences supporting the cerebellar involvement in cognitive processes and allows us to identify promising outcome measures for future trials focusing on cognitive dysfunction.

## Background

Spinocerebellar Ataxia type 2 (SCA2) is an autosomal dominant neurodegenerative disorder. The disease is caused by the expansion of the Citosine-Adenine-Guanine (CAG)-repeat tract in the coding region of the *ATXN2* gene, causing the expression of an abnormally long polyglutamine tract in the ataxin-2 protein which consequently acquires neurotoxic functions resulting in neuronal loss of the cerebellum, brainstem, brain cortex and spinal cord [1, 2]. Clinical features of SCA2 comprise a progressive cerebellar syndrome accompanied by a marked slowing of horizontal saccadic eye movements, sensory-motor peripheral neuropathy, pyramidal signs and cognitive dysfunction [3]. Most of the non-motor features of this disease antecede the ataxia onset by some years, defining it as the SCA2 prodromal stage [4].

Globally, SCA2 is the second most common autosomal dominant cerebellar ataxia, after SCA3, but is the most prevalent subtype in Mexico, Italy, India and Cuba. Where in Cuba, a founder effect of the SCA2 mutation results in the highest prevalence rates found worldwide [3, 5, 6]. Unfortunately, no effective neuroprotective treatments are currently available for this disease, nevertheless symptomatic treatments can improve some motor and non-motor features, but do not halt disease’s progression [3]. However, emerging findings of antisense oligonucleotides therapy in SCA2 mouse models represent a hopeful neuroprotective strategy for humans, which encourage the physicians and researchers to find better outcome measures [7]. Electrophysiological approaches have offered objective and reliable biomarkers for the evaluation of treatment’s feasibility and efficacy in various neurodegenerative conditions [8–10]. Among them, the event-related evoked potentials (ERP) have become in useful tools as electroencephalographic (EEG)-based methods for studying the brain’s synaptic function during cognitive processes in these diseases [11–15]. In particular, the P300 component of the ERP has been widely applied because it reflects the neurophysiologic substrate of cognitive processes such as attention, discrimination, and working memory [16].

Nevertheless, ERPs have not been systematically studied in SCA2, in spite of the early cognitive dysfunctions characterizing the disease [17]. Thus, in a previous study assessing the ERPs in SCA2 patients, Kremlacek et al., 2011 found significant associations of the ERPs deficits with the disease progression in a small cohort of Czech patients but there was not a preclinical carriers’ group within the study [18]. Therefore, the aim of this present research is to evaluate P300 component as biomarker of the SCA2 through a cross-sectional study in 30 patients and 20 preclinical carriers using visual and auditory P300 ERPs.

## Methods

### Subjects

#### SCA2 patients

Thirty clinically and genetically diagnosed SCA2 patients (11 females, 19 males) were admitted to the Centre for the Research and Rehabilitation of Hereditary Ataxias for this study. The mean age was 38.7 y/o (standard deviation [SD]: 11.2; range: 20–61), and the mean age of onset was 29.7 y/o (SD: 10.3; range: 15–53), whereas the disease durations varied from 1 to 19 years, with a mean of 8.60 (SD: 4.6). The number of CAG repeats ranged between 35 and 46 triplets (mean: 39.9; SD: 2.7) in the expanded alleles and between 21 and 25 triplets (mean: 22.1; SD: 0.6) in the unexpanded alleles.

#### SCA2 preclinical carriers

Together with the SCA2 patients, 20 SCA2 preclinical carriers with the SCA2 mutation (females 15, males 5) were also included. To be enrolled, the preclinical carriers had to carry at least an expanded allele in the *ATXN2* gene, with absence of cerebellar syndrome. Mean age of these subjects were 35.9 y/o (SD: 10.2; range: 20–58), whereas mean number of CAG repeats was 36.3 triplets (SD: 2.3; range 32–40) in the expanded alleles and 22.05 triplets (SD 0.39; range 21–23) in the unexpanded alleles. The probable age of onset ranged from 31 to 64 years (mean 44.13; SD 9.11) whereas the mean predicted time to ataxia onset was 8.3 years (SD: 10.9; range: − 7.1 – 28.1). The probable age of onset was estimated from each CAG repeat length using an exponential model (1171.583 x e^[− 0.091*CAG expansion size]^) obtained in a large population of Cuban SCA2 patients, [1] and the predicted time to ataxia onset was calculated by subtracting the chronological age from the estimated age of onset. Subjects with predicted negative times to ataxia onset were excluded from the analyses because these are subjects that should have developed the disease but have not done so yet.

#### Healthy controls

Thirty-three healthy subjects (females 16; males 17) not belonging to any SCA2 families with ages from 19 to 65 y/o (mean 36.4; SD 11.2) were randomly chosen as age-, sex- and education level-matched controls. So, all SCA2 patients and preclinical carriers had at least one control subject. All studied subjects had at least a 9th grade education level, whereas 22 SCA2 patients (73%) and 14 preclinical carriers (70%) had at least a 12th grade education level.

This study was approved by the Ethics Committee of the Centre for the Research and Rehabilitation of Hereditary Ataxias (Holguín, Cuba) and was conducted according to the declaration of Helsinki. Each subject gave written informed consent for participation in this study.

### Neurological assessments

All subjects were evaluated clinically following the standardized Mayo Clinic procedures for neurological examination [19] and a structured medical interview. Within the preclinical carriers, the search of clinical features was exhaustively performed following the clinical criteria defining the SCA2 prodromal stage [4]. The Scale for the Assessment and Rating of Ataxia (SARA) was applied to evaluate the cerebellar signs. This scale includes eight items (three for upright posture, one for speech and four for limb kinetic function), yielding a total score of 0 (no ataxia) to 40 (most severe ataxia) [20].

### Neuropsychological assessments

The Stroop Color-Word Interference test was applied in all SCA2 patients and preclinical carriers. This test consists of a color naming condition where subjects name the color of colored patches, and an interference condition where subjects are shown an array of color names printed in different colored inks. The analyzed parameters were the corrected time to complete this task, obtained by subtracting the time needed for the color naming condition from the time needed for the interference condition, as well as the number of interference errors [21].

### Event-related evoked potential assessments

#### Behavioral paradigms

Visual and auditory oddball paradigms were given to subjects in a sound-attenuated room using the *NEURONIC ESTIMULADOR COGNITIVO* software (V2.1.0.0, NEURONIC S.A, Havana, Cuba). The visual oddball paradigm employed an image with a picture of a horse as the standard stimuli and an image with the picture of a bicycle as target, with probabilities of 80 and 20% respectively. A total of 100 stimuli were presented in a 24-in. computer monitor following a pseudo-randomized sequence with stimulus duration of 500 ms and a fixed inter-stimulus interval of 1200 ms.

During the auditory two-tone oddball paradigm, 100 auditory stimuli were presented binaurally by loudspeaker at an approximately 65-dB sound pressure level. Standard stimuli (80% of probability) consisted of 500 Hz tones whereas target stimuli (20% of probability) consisted of 1500 Hz tones. All tones had 200 ms duration and were presented with a fixed inter-stimulus interval of 1200 ms in a pseudo randomized manner.

In both paradigms, the subjects were instructed to press the spacebar key on the keyboard in response to targets stimuli only. In order to check that each subject had correctly understood the task all participants were required to verbalize the appropriate instructions and a short practice trial (10 stimuli) was applied before the task began.

#### Electroencephalography recordings

EEG signals were recorded synchronized with the execution of the oddball paradigms in a NEURONIC MEDICID-5 EEG apparatus, using the 10/20 system. Ag/AgCl EEG electrodes were used and referenced to mastoids. EEGs were digitally filtered with a bandpass of 0.5–30 Hz and the sample frequency was 200 Hz. The signals were amplified with a gain of 1000.

#### ERP analysis

Analyses were performed using the NEURONIC ANALISIS DE PSICOFISIOLOGIA software (V3.0.3.0, NEURONIC S.A, Havana, Cuba). Epochs in the EEGs were automatically segmented at intervals of 100 ms pre-stimulus and 850 ms post-stimulus, respectively. Epochs with blinks, eye movements, excessive muscle activity, or amplifier blocking were removed through automatic analysis and visual inspection. Also, epochs with incorrect responses were discarded. After specific channels were selected (Cz and Pz), valid data were averaged off-line and represented graphically as latency (x-axis) vs amplitude (y-axis).

The P300 components were analyzed for the target stimuli and they were identified as the maximum positivity occurring between 300 and 650 ms post stimulus onset and their peak latency was defined as the time point of greatest positive amplitude within the specific latency window. The amplitude of P300 was defined as the maximum positive voltage measured from baseline. Both latency and amplitude data were obtained for each electrode site individually. P300 abnormalities were considered when the P300 peak latencies were more than the 2 SD threshold of healthy controls’ mean latencies and/or P300 amplitudes were less than the 2SD of the healthy control’s mean amplitudes.

### Molecular studies

DNA extracts from peripheral venous blood underwent the amplification of CAG-rich region in the *ATXN2* gene by polymerase chain reactions with the previously published oligonucleotide primers [22] and determination of CAG repeats on polyacrylamide gel electrophoresis on an ALF Express II apparatus (Amersham Biosciences, Sweden).

### Statistical analyses

For descriptive statistics of quantitative variables, means and standard deviations were calculated. The normality of the distribution of quantitative variables was assessed by the Kolmogorov-Smirnov test. Mixed analyses of variance (mixed-ANOVA) followed by the Tukey’s HSD post-hoc tests were performed with the between-subject factor of groups (SCA2 patients, SCA2 preclinical carriers and healthy controls) and the within-subject factor of electrode site (Cz and Pz). Stepwise multiple regression models were performed to assess the influence of demographic, clinical and molecular parameters on the P300 variables. Common independent variables in the regression models of patients and preclinical carriers were the age, educational level, SARA score as well as unexpanded and expanded *ATXN2* allele sizes, whereas age at onset and disease duration were specifically included in the SCA2 patients’s regression model and the predicted time to ataxia onset was only analyzed in the preclinical carriers. Frequency analysis of P300 abnormalities was conducted using the Chi-square test (*X*^*2*^) followed by the Yates correction. Receiver operating characteristic (ROC) analyses were performed for each P300 variable as patients and preclinical carriers as positive level independently. All analyses were performed using the commercially available STATISTICA software package (StatSoft, Inc., 2003 STATISTICA data analysis software system, version 6. http://www.statsoft.com/Products/STATISTICA-Features).

## Results

### Clinical findings

Aside from the cerebellar syndrome, the most frequent disease features of SCA2 patients were the slowing of saccade eye movements (93.3%) and sensory abnormalities (83.3%). Age at onset was significantly correlated with the expanded CAG repeat (r = − 0.78; *p* < 0.00001). Mean SARA score was 13.01 (SD 5.22; range: 6–28). Within the preclinical carriers, the most frequent prodromal features were the painful muscle cramps (75%), sensory abnormalities (60%) and hyperreflexia (55%). Mean SARA score in this group was 0.23 (SD 0.45; range: 0–1.5).

### P300 peak abnormalities in SCA2

**Fig. 1:**
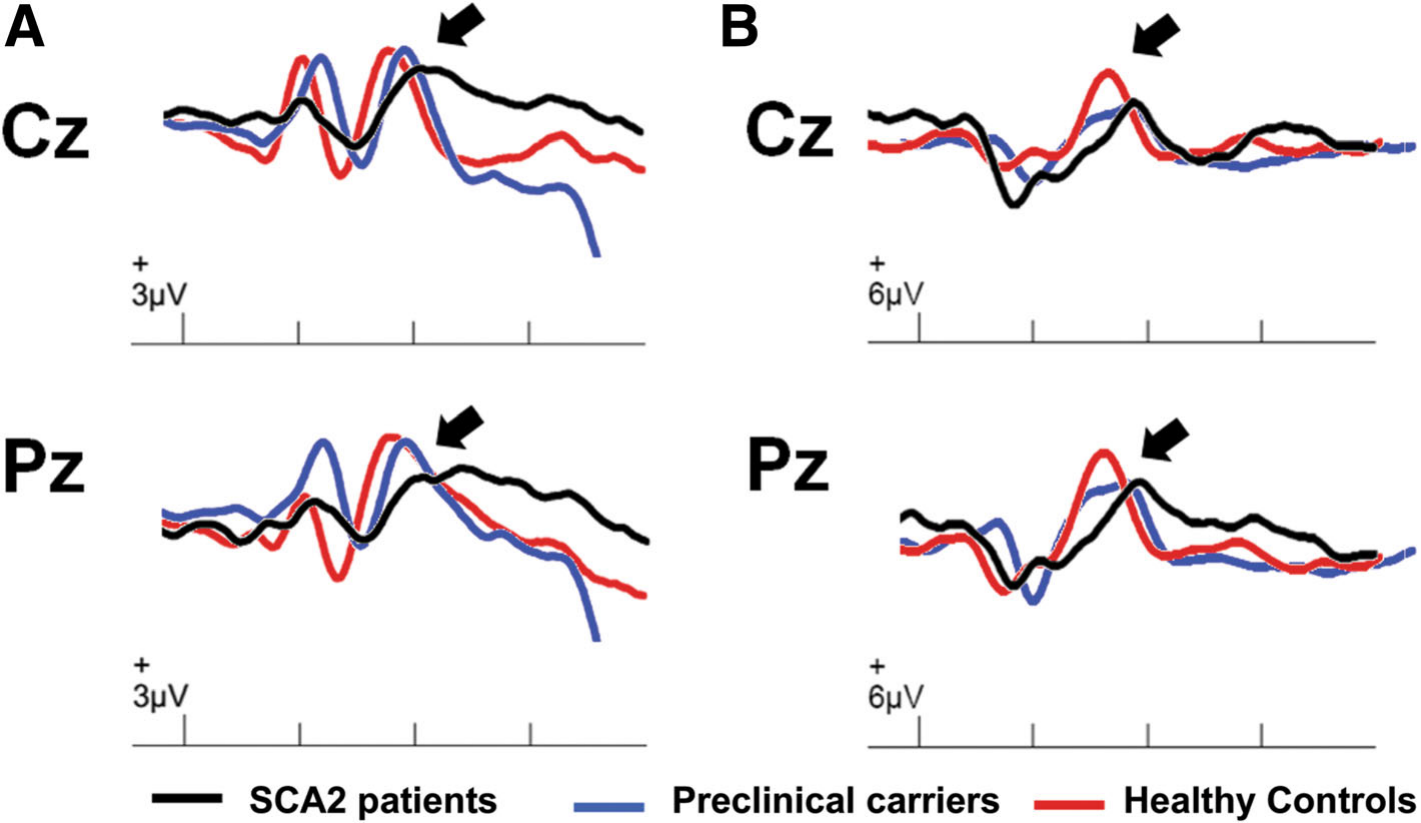
Grand averaged ERPs for visual (**a**) and auditory stimuli (**b**) from the target stimuli at Cz and Pz. Black trace: SCA2 patients; Blue trace: Preclinical carriers; Red trace: Controls

The grand average ERP waveforms for the visual and auditory stimulus conditions at Cz and Pz is shown in the Fig. 1. Mixed-ANOVAs disclosed a significant GROUP effect for all ERP variables, but no ELECTRODE SITE effect nor GROUP X ELECTRODE SITE interaction effect were observed. Tukey’s HSD post-hoc tests revealed that the visual and auditory P300 peak latencies and amplitudes were significantly different between SCA2 patients and controls at both electrode sites, whereas in the preclinical carriers group only the visual P300 peak latency at Cz and auditory P300 peak latency at Pz were prolonged when compared to the control group. In addition, the visual P300 peak latencies at Cz and Pz as well as the auditory P300 peak latency at Cz were statistically different between SCA2 patients and preclinical controls (Table 1).

**Table 1:** Mixed-ANOVA of P300 variables for SCA2 patients, preclinical carriers and healthy controls

ERP	Variable	SCA2 patients	Preclinical carriers	Healthy controls	Mixed-ANOVA		
					Group	Site	Group x Site
Visual	Latency						
	Cz	462.80 ± 7.40***^/¶¶^	421.34 ± 9.06*	384.68 ± 7.08	F = 53.73	F = 0.12	F = 0.10
	Pz	463.41 ± 7.40***^/¶¶^	420.46 ± 9.92^ns^	390.54 ± 8.10	*p* < 0.0001	*p* > 0.05	*p* > 0.05
	Amplitude						
	Cz	9.23 ± 1.12**	11.63 ± 1.41^ns^	15.28 ± 1.10	F = 14.00	F = 0.20	F = 0.06
	Pz	9.03 ± 1.15**	11.44 ± 1.43^ns^	14.39 ± 1.09	*p* < 0.0001	*p* > 0.05	*p* > 0.05
Auditory	Latency						
	Cz	437.91 ± 8.88***^/¶¶^	380.66 ± 10.88^ns^	358.05 ± 9.64	F = 41.18	F = 1.09	F = 0.61
	Pz	438.21 ± 9.79***	401.67 ± 11.79*	360.94 ± 10.76	*p* < 0.0001	*p* > 0.05	*p* > 0.05
	Amplitude						
	Cz	9.47 ± 1.25*	12.63 ± 1.51^ns^	16.99 ± 1.29	F = 9.47	F = 0.263	F = 0.03
	Pz	9.48 ± 1.11*	11.48 ± 1.33^ns^	15.50 ± 1.22	*p* < 0.0001	*p* > 0.05	*p* > 0.05

Asterisks denote the statistical differences between SCA2 patients or preclinical carriers and controls (*:*p* < 0.05; **:*p* < 0.005; ***:*p* < 0.0005). Pilcrow denote the statistical differences between SCA2 patients and preclinical carriers (^¶¶:^ *p* < 0.005)

**Fig. 2:**
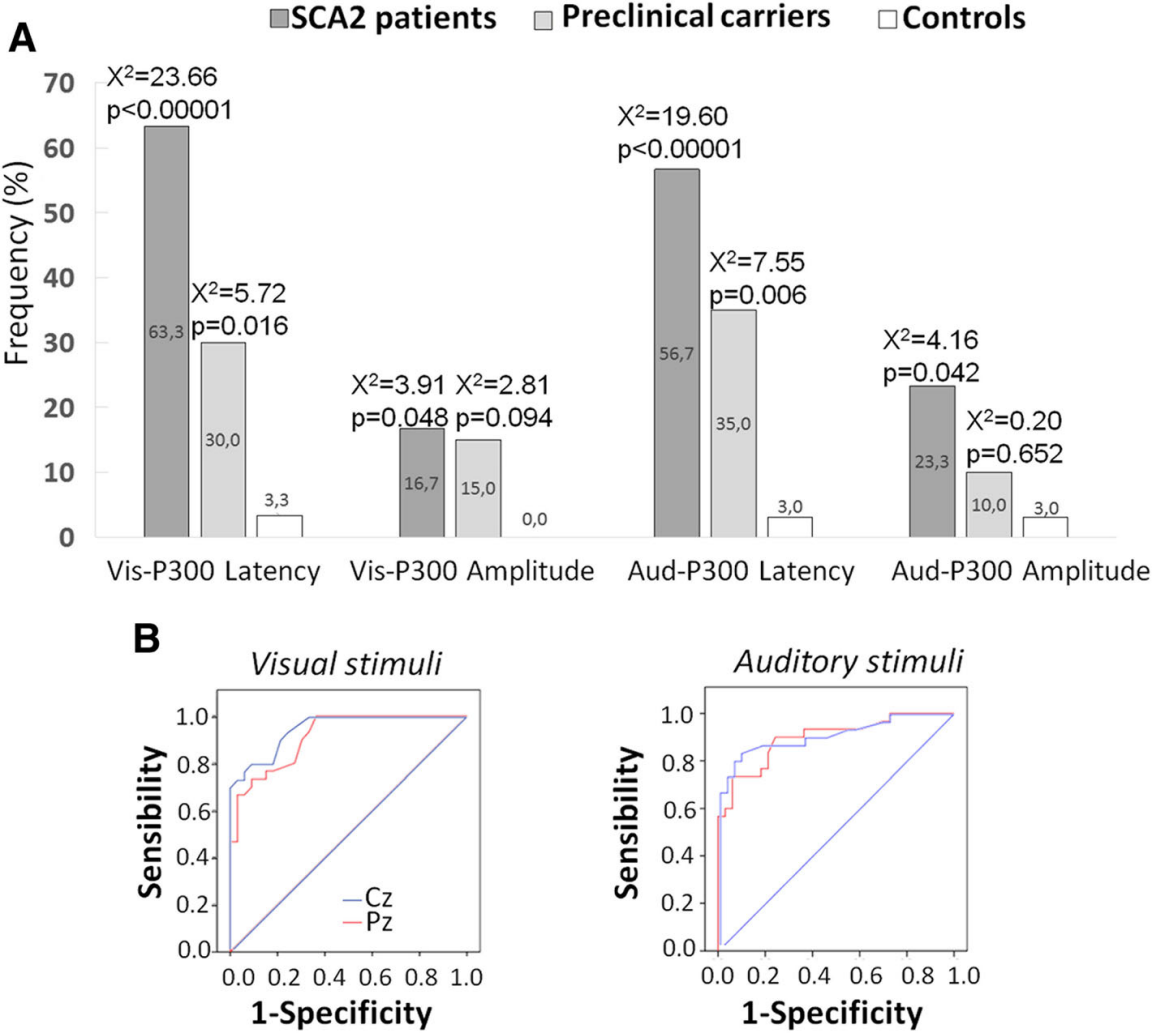
Frequency analysis of P300 abnormalities in SCA2 patients and preclinical carriers and controls (**a**) and ROC analysis of P300 peak latency abnormalities in SCA2 patients (**b**). Blue trace: Cz; Red trace: Pz

The frequency analyses revealed a significant higher proportion of SCA2 patients with P300 abnormalities, as well as a higher proportion of SCA2 preclinical carriers with prolonged P300 peak latencies (Fig. 2a). The ROC analysis disclosed higher area under the curve for the P300 peak latencies in SCA2 patients both for visual (Cz: 94.8; Pz: 91.3) and auditory stimuli (Cz: 91.2; Pz: 89.7) (Fig. 2b). The remaining areas under the curves were below 80%.

### Influence of demographical, clinical and molecular variables on P300 peak abnormalities

**Fig. 3:**
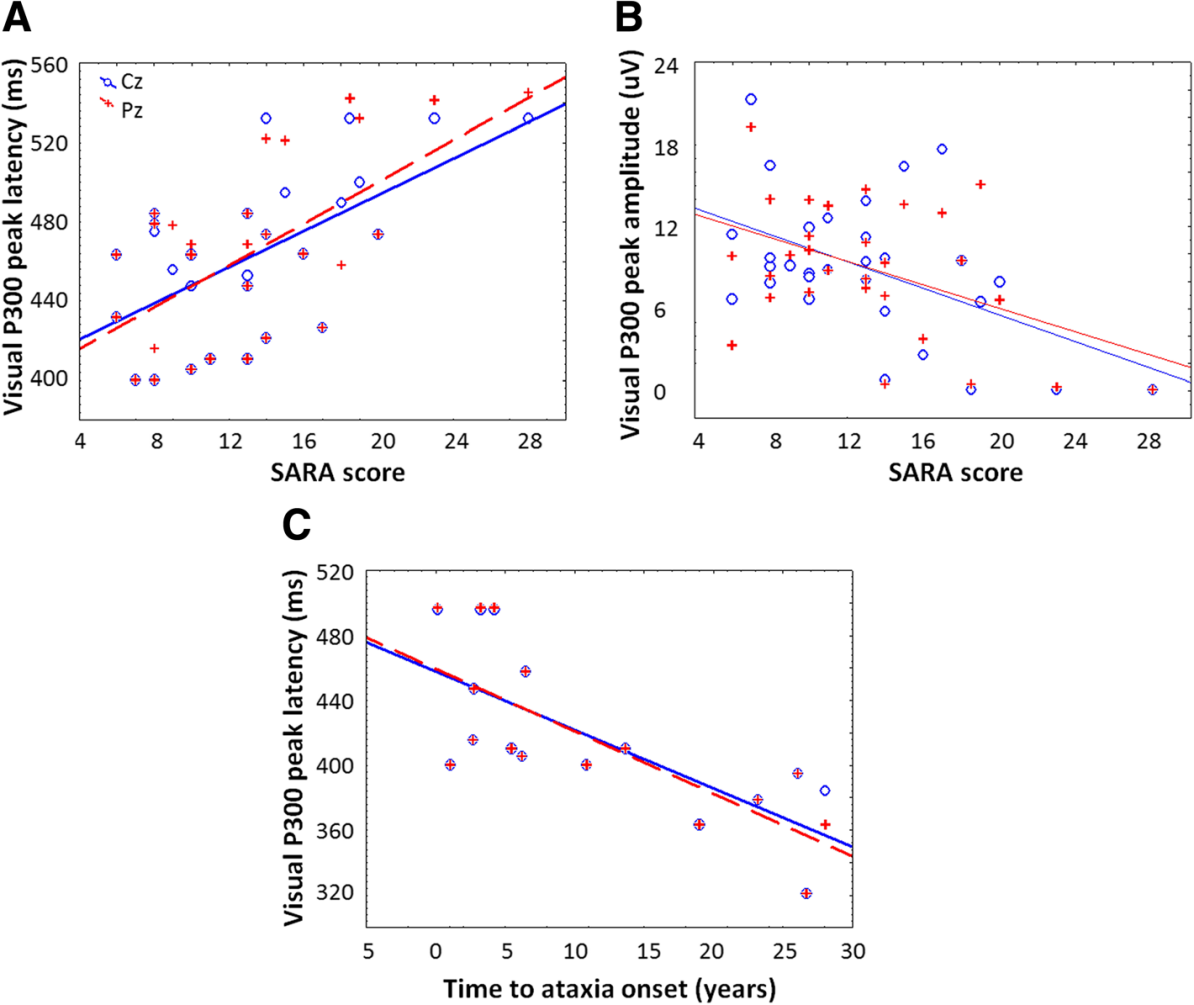
Scatterplots of P300 parameters vs clinical and demographical variables in SCA2 patients and preclinical carriers. **a**: Visual P300 latencies vs SARA score in SCA2 patients; **b**: Visual P300 amplitudes vs SARA score in SCA2 patients; **c**: Visual P300 latencies vs time to ataxia onset in preclinical carriers

Multiple regression analyses within the SCA2 patients group identified the SARA score as the main determinant of the visual P300 latencies (Cz: β = 0.48, *p* = 0.013; Pz: β = 0.63; *p* = 0.001) and amplitudes (Cz: β = − 0.51; *p* = 0.012; Pz: β = − 0.48; *p* = 0.013). Within preclinical carriers, significant standardized regression coefficients were observed between the predicted time to ataxia onset and the visual P300 peak latency (Cz: β = − 0.71; *p* = 0.003; Pz: β = − 0.74; *p* = 0.002). Scatterplots of these regressions were shown in the Fig. 3. Correlation analyses in healthy controls disclosed significant association between age and visual P300 latency (r = − 0.49; *p* = 0.006).

### Relationship between P300 peak variables and Stroop test performance

Correlation analyses between the P300 parameters and the Stroop test performance variables are shown in Table 2. After the Bonferroni correction, the Stroop interference time correlated with the visual and auditory P300 latencies in SCA2 patients and with the visual P300 peak latencies in the preclinical carriers. The number of Stroop interference errors showed no correlation with the P300 variables.

**Table 2:** Findings of correlation analyses between P300 variables and the interference Stroop-test performance in SCA2 patient and preclinical carriers

Variables	SCA2 patients				Preclinical carriers			
	Stroop interference time		Stroop interference errors		Stroop interference time		Stroop interference errors	
	r	p	r	p	r	p	r	p
V-Lat Cz	0.50	0.006	0.25	0.209	0.63	0.003	-0.13	0.582
V-Amp Cz	-0.23	0.246	-0.10	0.624	-0.48	0.034	-0.03	0.890
V-Lat Pz	0.51	0.006	0.22	0.259	0.64	0.003	-0.12	0.618
V-Amp Pz	-0.26	0.181	-0.13	0.500	-0.47	0.035	-0.04	0.865
A-Lat Cz	0.56	0.002	0.31	0.108	0.48	0.031	0.22	0.364
A-Amp Cz	-0.38	0.046	-0.20	0.309	-0.48	0.034	-0.05	0.840
A-Lat Pz	0.61	0.001	0.32	0.094	0.19	0.414	-0.05	0.819
A-Amp Pz	-0.42	0.025	-0.22	0.257	-0.17	0.477	0.25	0.284

## Discussion

The present paper shows the most extensive study of ERP potentials in SCA2 or any other SCA and it demonstrates that the P300 component abnormalities antecede the ataxia onset by some years and are closely associated to the progression of the cerebellar syndrome. In general, P300 component abnormalities reflect impaired processes related to attention, decision making and memory updating [16, 23]. Particularly, the P300 latency is considered as an index of the processing time required before response generation, whereas the P300 amplitude is thought to reflect the brain activity required to maintain working memory during the stimulus context updating [16, 24]. Therefore, the P300 abnormalities observed in SCA2 reflect the impairment of the brain activity underlying the processing of incoming information and cognitive processes such as attention and working memory.

This assertion was corroborated in the present paper by the close association between the P300 latencies and Stroop test performance in SCA2 patients and preclinical carriers. Also, these correlations support the validity of ERPs in evaluating the cerebellar cognitive-affective syndrome (CCAS) in SCA2, although other studies using other conventional neurocognitive assessments, e.g. the Schmahmann syndrome scale, are mandatory.

The higher areas under the curve obtained from the P300 peak abnormalities in SCA2 patients suggest the substantial clinical value of these electrophysiological methods as a tool to complement the disease diagnosis and to study the cognitive decline in these patients. Interestingly, we found that visual P300 abnormalities were more accentuated in SCA2 patients with higher SARA scores, suggesting that the cognitive processes underlying this psychophysiological response are impaired alongside the motor deterioration in SCA2 patients. These findings are in concordance with a previous work carried out in a distinct cohort of Cuban SCA2 patients, which showed significant associations between SARA scores and the performance of automated cognitive tests assessing attention and visual memory [25]. Moreover, in a 2-year follow-up study performed in 22 Italian SCA2 patients, Fancellu et al., 2013 found that the SARA score and attentional deficits worsened significantly during the follow-up period while other cognitive domains did not become impaired [26]. In addition to, the relationships between P300 abnormalities and SARA scores could reflect the individuals’ awareness of motor worsening. However, there are not any studies supporting the role of the illness’ awareness on the P300 parameters in movement disorders, which set the rationale for further investigations about this issue.

The presence of P300 abnormalities in prodromal SCA2 suggests that the abnormalities of the stimulus information processing appear some years before the ataxia onset, which coincides with previous works showing the cognitive deterioration in prodromal SCA2 patients [27]. In particular, the visual P300 latency showed a significant association with the time before ataxia onset. Thus, SCA2 preclinical carriers with higher probabilities to be clinically diagnosed with the cerebellar syndrome had larger P300 latencies. This finding suggests that P300 abnormalities progresses insidiously during the prodromal stage of SCA2 patients, identifying this variable as preclinical biomarker of the disease.

Several studies have demonstrated that P300 abnormalities increase with aging during the adulthood [28], which was corroborated in our healthy control and preclinical carrier cohorts, but not in the SCA2 patients, likely due to other influencing factors that modifies the relationship between age and P300 variables in the patients.

Abnormalities of P300 parameters in SCA2 patients can be explained by the degeneration of some sites involved in the generation of this potential [15, 29, 30] such as the frontal cortex, thalamus, amygdala, hippocampus and basal ganglia. Furthermore, the P300 abnormalities in SCA2 could result from the cerebellar degeneration. This assertion is supported by a meta-analysis that demonstrated the activation of the left cerebellum during the oddball stimulus processing [31]. Moreover, Rusiniak et al., 2013 reported fMRI activations of the cerebellum during the registration of ERP elicited by an oddball paradigm in children [32]. Also, the transcranial direct current stimulation over the left cerebellum in healthy subjects reduce the P300 amplitudes for both the target and novel stimuli, suggesting a role of the cerebellum in the generation of this ERP [33].

In addition to these evidences, the P300 impairment in subjects with chronic and acute cerebellar lesions supports our findings and suggests a putative role of this structure in the P300 component generation. On this regards, in 1997, Mochizuki et al., reported the prolongation of P300 latency in a group of 10 cases with olivopontocerebellar atrophy [34], whereas Kamitani et al., 2001 found a significant association between the P300 abnormalities and MRI cerebellar size in a cohort of 15 patients affected by multiple system atrophy with predominant cerebellar features [35]. As noted above, Kremalcek et al., 2011 reported visual P300 abnormalities in a small cohort of SCA2 patients, with significant correlations of these electrophysiological impairments with the duration and progression of the disease [18]. Moreover, a reduction in auditory P300 amplitude was observed in a 55-year-old man with acquired ataxia after an acute ischemic lesion in the left posterior cerebellar hemisphere. These findings were interpreted as disruption of attentional resources mediated by a cerebello-cerebral diaschisis [36].

Thus, we could hypothesize that the cerebellar involvement in SCA2 patients could likely alter P300 components by interfering with attentional and working memory processes as result of the dysfunction of the cerebellar projections to prefrontal and posterior-parietal cortices [37–39], which are involved in these cognitive processes.

P300 abnormalities have also been reported in manifest and pre-manifest subjects with Huntington disease (other polyglutamine disease), which suggest the vulnerability of the P300 generation network to the polyglutamine expansions regardless of specific mutated proteins [14, 40] Nevertheless, the studies of P300 abnormalities in other Polyglutamine diseases are mandatory to confirm this. Similarly to our findings, abnormal P300 parameters have been identified in carriers of gene mutations that lead to familial Alzheimer disease [41–43] and have been considered a useful marker for monitoring the process through which Mild Cognitive Impairment becomes Alzheimer disease [43–45].

Limitations of this study include the unavailability of imaging measures which could be useful to understand the neuroanatomical base of P300 abnormalities in SCA2. Also, we cannot distinguish between the P3a and P3b components of the EPR which impedes our knowledge about the involvement of frontal and temporo-patietal mechanisms underlying attention [23]. Nevertheless our findings have impacts for future directions in these patients. For example, the combined use of P300 abnormalities with other electrophysiological biomarkers, neuroimaging and biochemical markers could aid in obtaining a better patient stratification and/or to predict phenoconversion.

## Conclusions

In summary, our paper demonstrated consistently the presence of P300 abnormalities in SCA2 patients and preclinical carriers, thereby giving support for the usefulness of this ERP in the search of surrogate biomarkers for cognitive deterioration. ERP provides a potential value for the prediction of the SCA2 disease’s onset and progression as well as for the study of cerebellar involvement in cognitive processes. Consequently, these psychophysiological variables represent promising outcome measures for future trials focusing on cognitive dysfunction.

## Abbreviations

ANOVA: Analysis of Variance
*ATXN2*: *Ataxin-2*
CAG: Cytosine-adenine-guanine
ERP: Event-related potentials
fMRI: Functional Magnetic Resonance Imaging
ROC: Receiver Operating Characteristic
SARA: Scale for the Assessment and Rating of Ataxia
SCA2: Spinocerebellar Ataxia type 2
SD: Standard Deviation
